# Streptococcus sanguinis Endocarditis of Bicuspid Aortic Valve Presenting as Septic Arthritis of Lumbar Facet Joint

**DOI:** 10.7759/cureus.24189

**Published:** 2022-04-16

**Authors:** Pranitha Kovuri, Sriviji Senthil Kumaran, Tulika Chatterjee

**Affiliations:** 1 Internal Medicine, University of Illinois College of Medicine, Peoria, USA

**Keywords:** bicuspid aortic valve disease, infective endcardititis, septic arthritis, lumbar facet joint, streptococcus sanguinis, low back pain, safj

## Abstract

Septic arthritis of the facet joint (SAFJ) is an uncommon etiology of low back pain that usually affects the elderly population and immunocompromised patients but is rare in immunocompetent and young patients. When such a clinical presentation occurs, it is imperative to diagnose the source of the infection. We report a case of septic arthritis of the left third and fourth lumbar vertebrae facet joint due to *Streptococcus sanguinis *in a young immunocompetent adult, and the source of infection was found to be subacute infective endocarditis of a bicuspid aortic valve which was undiagnosed till now. A 49-year-old male presented with new-onset palpitations, dyspnea with exertion, low back pain, night sweats, and chills. A physical exam was significant for spinal tenderness on palpation of the lumbar spine around the L3-L5 level. Blood cultures were positive for *Streptococcus sanguinis*, and an MRI of the lumbar spine showed left-sided L3-L4 septic arthritis with epidural abscess and posterior paravertebral cellulitis/myositis. Transesophageal echocardiography led to the diagnosis of a bicuspid aortic valve and moderate aortic insufficiency, but it was a cardiac computed tomography that showed a sub-aortic valve abscess leading to the diagnosis of infective endocarditis. He was treated with a six-week course of intravenous antibiotics with complete resolution of symptoms, followed by aortic valve replacement with a mechanical valve. This case report focuses on the importance of diagnosing occult sources in clinically atypical infections, especially when hematogenous seeding is suspected.

## Introduction

Low back pain (LBP) is one of the most common chief complaints of patients presenting to outpatient clinics and emergency rooms (ER). Young individuals usually present with ligament sprains, muscle strains, or herniated discs. Septic arthritis of the facet joint (SAFJ) is an uncommon etiology of LBP. It usually affects the elderly population and immuno-compromised patients, but there are cases of SAFJ reported in immuno-competent and young patients [1]. We report a case of septic arthritis of the left lumbar L3-L4 facet joint due to streptococcus sanguinis in a healthy 49-year-old male. *Staphylococcus aureus* is the organism most commonly implicated in cases of SAFJ, but *Streptococcus* species is rarely reported as an etiology of SAFJ [2]. *Streptococcus sanguinis* is an organism well known to cause infective endocarditis [3]; thus, we investigated endocarditis in our patient. The transesophageal echocardiography (TEE) revealed a bicuspid aortic valve previously missed on transthoracic echocardiography (TTE). Cardiac computed tomography (CT) revealed the presence of a subvalvular abscess which could not be identified on the TTE or TEE. Thus, the etiology of *Streptococcus sanguinis* SAFJ was identified in our patient as septic emboli from infective endocarditis of the bicuspid aortic valve.

When primary care physicians or internists come across such unusual presentations, it is important to consider occult sources of infections. Subacute infective endocarditis usually presents with nonspecific symptoms and requires a high index of suspicion for early diagnosis. Perivalvular abscesses should remain high on the differential when patients suspected to have infective endocarditis present with conduction abnormalities. 

## Case presentation

A 49-year-old male with a past medical history of hypertension and hyperlipidemia presented to the outpatient clinic with a chief complaint of chest-pounding, palpitations, generalized weakness, and LBP ongoing for three weeks. He reported that he was moving heavy objects right before the onset of his back pain. He also reported chills associated with painful rigors, night sweats, and worsening generalized fatigue hampering his daily activities. He had noted that he was becoming short of breath with exertion and experienced chest pain with deep breaths. He denied any tingling, numbness of extremities, bowel or bladder incontinence, rhinorrhea, congestion, cough, sore throat, nausea or vomiting, abdominal pain, diarrhea, urinary symptoms, or weight loss. He had no history of intravenous drug abuse or any other substance abuse. He had no history of sick contacts, recent travels, exposure to wooded areas, or bug bites. An electrocardiogram (EKG) was done at the outpatient clinic, which showed a Mobitz type 1 atrioventricular (AV) block.

**Figure 1:**
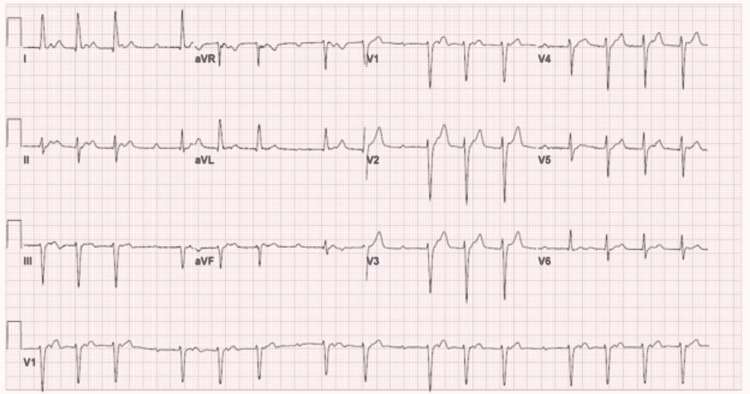
Admission EKG Showing sinus tachycardia with second degree AV block (Mobitz type 1) AV - atrioventricular

On presentation, he was febrile with a temperature of 100.5 F, tachycardic with a heart rate (HR) of 91 beats/min, and blood pressure (BP) of 142/69 mm Hg. A physical exam was significant for spinal tenderness on palpation of the lumbar spine around the L3-L5 level. Initial laboratory workup revealed white blood cells (WBC) count of 16,750/mcL with 78.6% neutrophils, hemoglobin (Hb) of 12.6 g/dL, normal electrolytes, normal lactic acid, and creatinine. The patient had an elevated erythrocyte sedimentation rate (ESR) of 77 mm/h and C-reactive protein (CRP) of 16.25 mg/dL. Repeat EKG showed sinus tachycardia with second-degree type I AV block and poor R wave progression (Figure 1).

Troponin was normal, and D-dimer was elevated to 0.98. Chest X-ray and computed tomographic (CT) angiogram of the chest did not show any abnormalities. Broad-spectrum antibiotics were started with intravenous vancomycin and piperacillin/tazobactam. Transthoracic echocardiography (TTE) showed normal left and right ventricular function and normal bilateral atria. Overall, imaging quality was poor, and the aortic valve was thought to appear tricuspid with moderate eccentric aortic regurgitation, and the aortic root was dilated at the sinuses of Valsalva.

**Figure 2:**
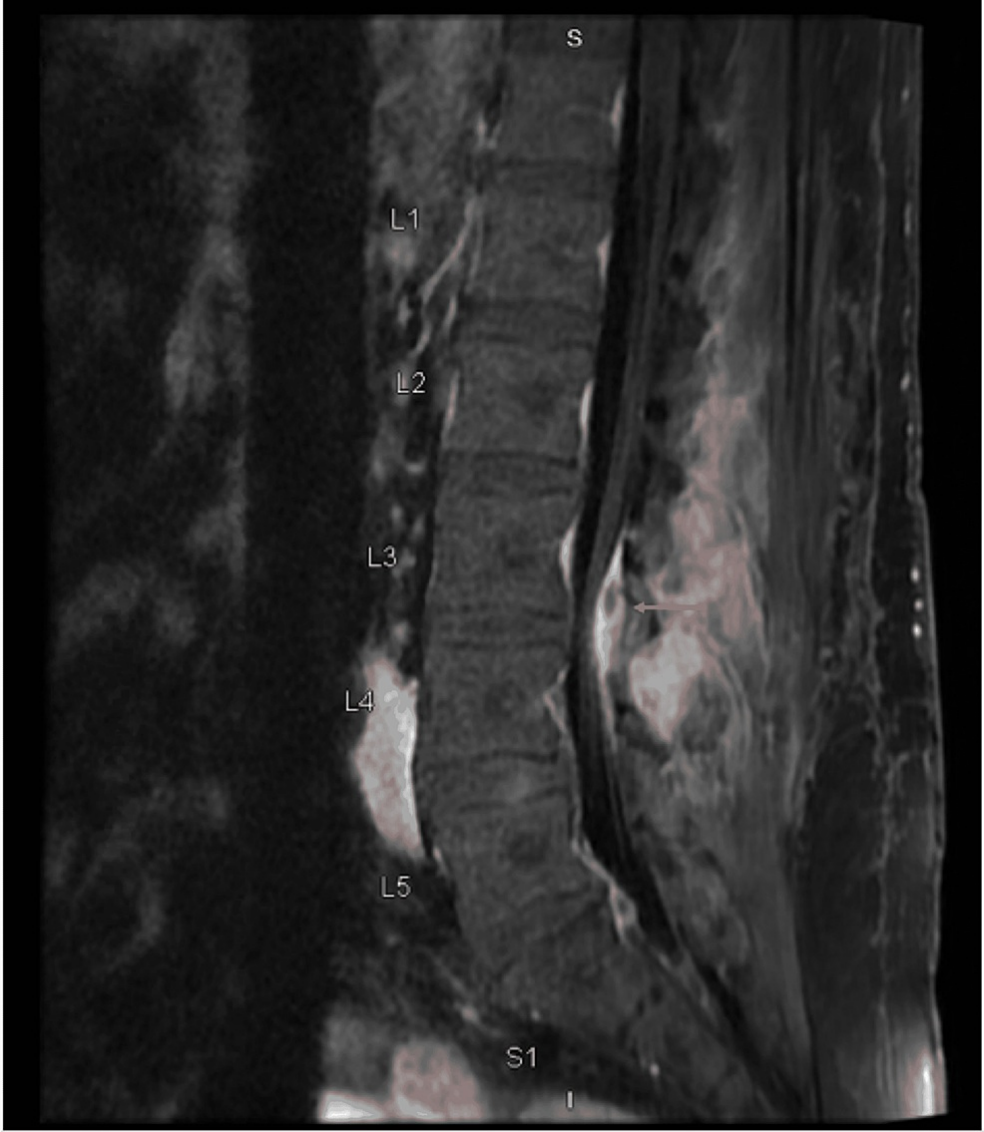
T1 flair sagittal section showing L3-L4 facet joint septic arthritis with epidural abscess (arrow) and posterior para-vertebral cellulitis/myositis

**Figure 3:**
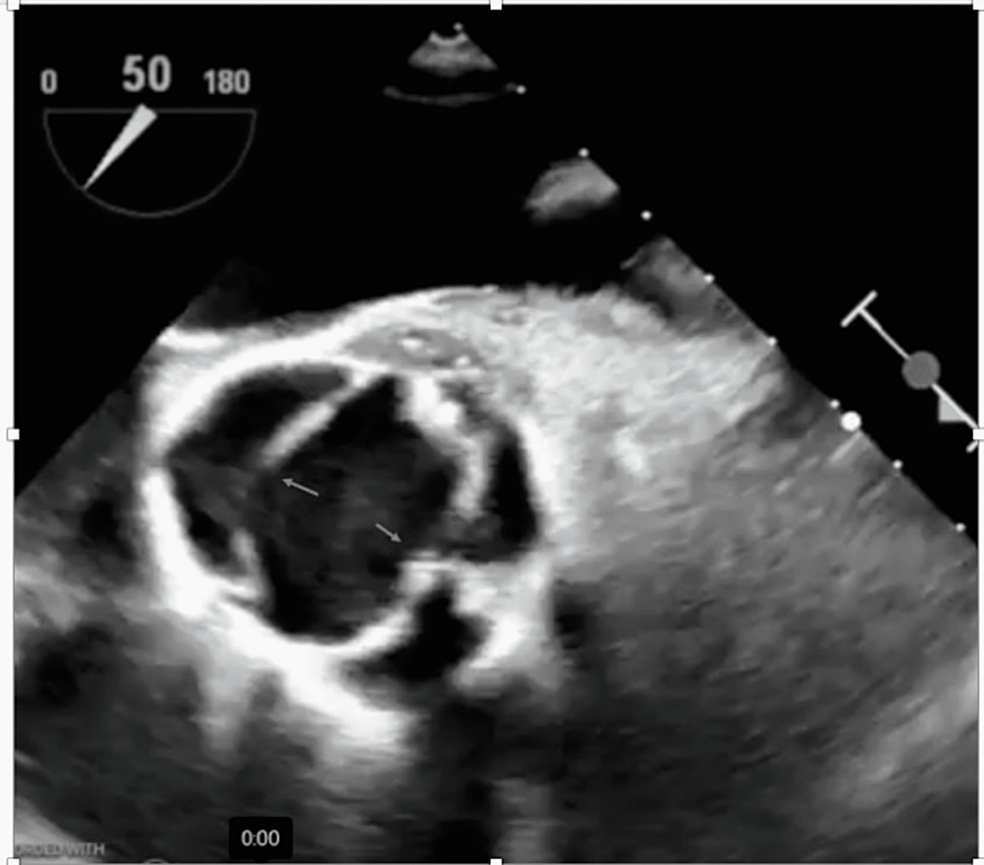
Transesophageal echo showing aortic valve with two leaflets (arrows) in the open position confirming bicuspid aortic valve in the short-axis view

**Figure 4:**
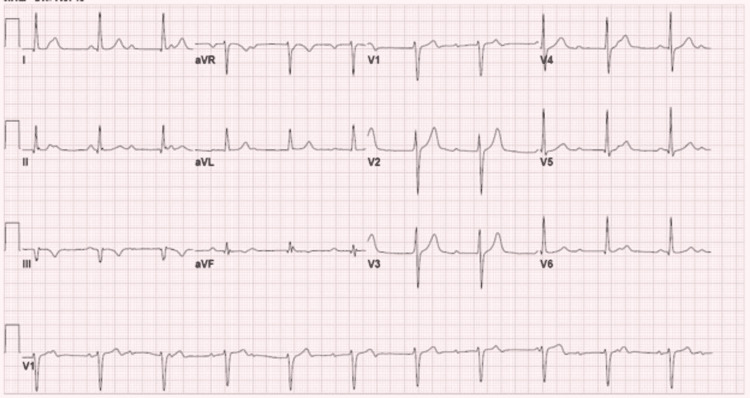
EKG documenting intermittent complete heart block and junctional escape rhythm

**Figure 5:**
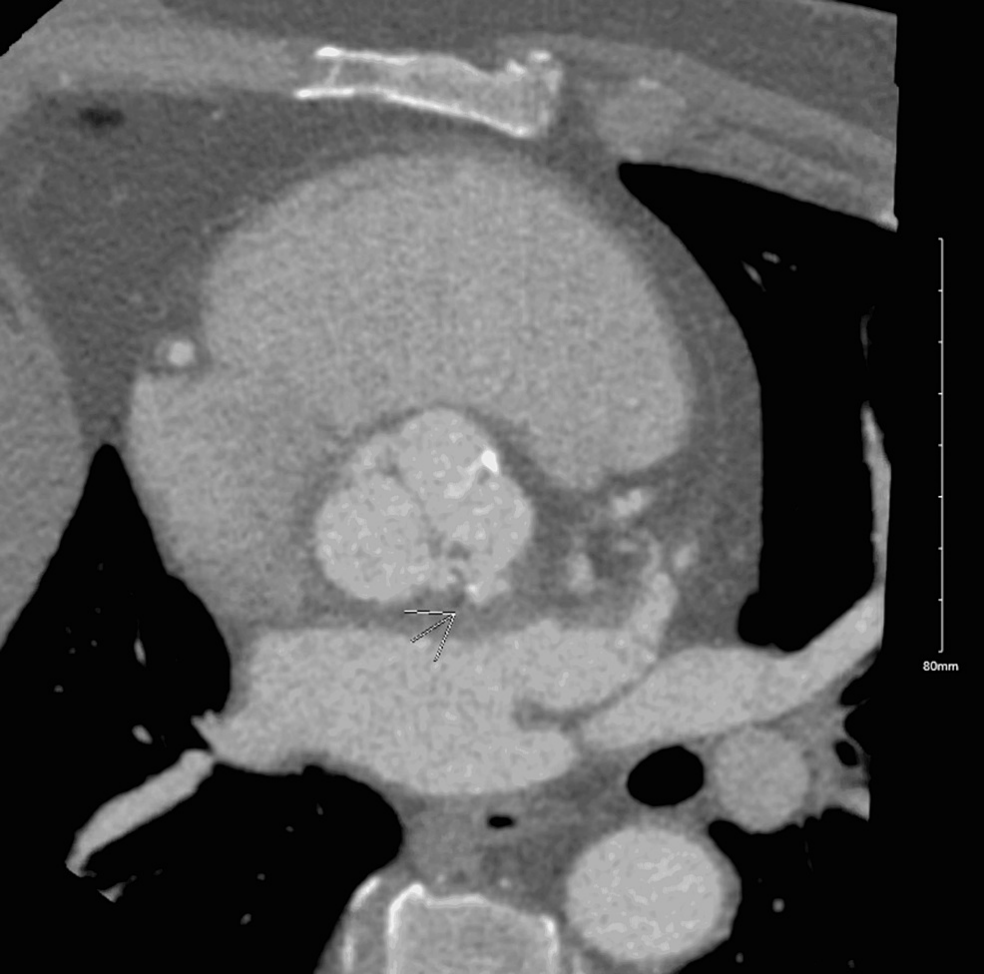
Cardiac axial section CT at the level of the aortic valve with an arrow showing peri-valvular abscess

Broad-spectrum antibiotics were started with intravenous vancomycin and piperacillin/tazobactam. X-ray panorex showed no definite evidence of periapical abscess. Blood cultures were positive for *Streptococcus sanguinis*. Antibiotics were changed to intravenous ceftriaxone based on the susceptibilities. MRI of the lumbar spine showed septic arthritis of left-sided L3-L4 facet joints with epidural abscess and posterior paravertebral cellulitis/myositis (Figure 2). Neurosurgery recommended medical management and offered no surgical intervention. Transesophageal echocardiography (TEE) was now performed to rule out infectious endocarditis in the setting of *Streptococcus sanguinis* bacteremia, which showed normal ejection fraction, no vegetations but revealed bicuspid aortic valve with moderate to severe aortic insufficiency (Figure 3, Video 1). The bicuspid aortic valve was missed in the previous TTE. Serial EKGs were done during hospitalization, which showed a high degree of AV block with intermittent complete AV block (Figure 4), but he was not transferred to the intensive care unit as he remained hemodynamically stable. Given the progression of the patient's rhythm from second-degree type 1 AV block to intermittent complete heart block, cardiac CT was done, which showed a small aortic root pseudoaneurysm, which was secondary to the perivalvular abscess (Figure 5). The left coronary cusp was perforated with severe aortic insufficiency. The patient was thus diagnosed with infective endocarditis in the setting of an undiagnosed bicuspid aortic valve. A six-week course of ceftriaxone was prescribed for L3-L4 septic arthritis with epidural abscess and aortic valve endocarditis, following which the patient underwent successful mechanical aortic valve replacement for severe aortic insufficiency.

**Video 1:** Transesophageal echo showing bicuspid aortic valve with moderate aortic regurgitation (short-axis view)

## Discussion

Septic arthritis commonly affects large peripheral joints like knees, hips, ankles, shoulders, elbows, and wrists. It rarely affects small joints like sternoclavicular joints, pubic symphysis, and spinal facet joints [4]. Haplin et al. reported the first case of septic arthritis of the facet joint in 1987 [5]. The most common site of SAFJ is the lumbar spine, but there are a few reported cases of SAFJ involving the cervical spine and thoracic spine [6,7]. Even though there has been a recent increase in case reports of SAFJ due to improvements in the imaging techniques, the true incidence of SAFJ is unknown [4,6].

The most common microorganism associated with the septic joint is *Staphylococcus aureus*. *Streptococcus *species, gram-negative bacilli, and *Enterococcus faecalis* can also cause SAFJ but with much lesser frequency [8]. Among the *Streptococcus *species, beta-hemolytic *Streptococcus *species like *S. pyogenes* and *S. agalactiae* are more commonly implicated in causing septic arthritis. Alpha hemolytic species, viridans (mutans, sanguinis), are rarely associated with bone and joint infection [9,10].

Pathogenesis of SAFJ is likely due to hematogenous spread from a distant nidus of infection [6]. In a retrospective review of around 190 cases of septic arthritis, it was found that 72% of cases were due to hematogenous spread of infection [7]. Contiguous spread of infection from adjacent structures like discs or muscles or vertebral bodies can rarely occur [5]. In our patient, SAFJ septic arthritis occurred due to hematogenous spread from the perivalvular abscess of the aortic valve, which was incidentally found to be bicuspid.

The bicuspid aortic valve is common congenital heart disease and has an incidence of almost 1% in the general population [11]. The presentation and diagnosis of this condition are highly heterogeneous and, therefore, can be diagnosed at any time from birth to older individuals. It can be a benign presentation as in a patient who comes in for a routine visit found to have the characteristic click and murmur vs. early severe valve dysfunction, premature congestive heart failure, thoracic aortic aneurysms, and infective endocarditis, all of which are common complications associated with this pathology [11].

Tribouilloy et al. performed a multicenter observational study in France to investigate the characteristics and outcome of infective endocarditis (IE) in adults with bicuspid aortic valves (BAV) [12]. They noted that BAV IE occurred more frequently in a younger population and was more frequently complicated by severe valvular and perivalvular complications. Perivalvular abscesses were identified in about 50% of BAV IE patients. Another study by Lamas and Eykyn reported a 30% incidence of the same [13]. Both studies identified *Streptococcus *as the most common pathogen. Of note, perivalvular abscesses occur in about 30-40% of the patients with infective endocarditis. The aortic valve, in general, is more susceptible to abscess formation. As these abscesses extend, they involve the conduction tissues and present with different types of AV block. A new onset AV block in the presence of infective endocarditis/ persistently positive blood cultures should raise the suspicion of perivalvular abscess [14].

TTE is the first line of investigation in all patients suspected to have infective endocarditis. However, TTE has low sensitivity but high specificity if vegetations are identified. Diagnosis of the bicuspid aortic valve can itself be challenging despite well documented echocardiographic diagnostic features. It has been reported that up to 10-15% of the individuals continue to have a diagnostic uncertainty despite TTE with good quality imaging [15]. Our patient had poor quality TTE images, and in addition, a reliable M-mode could not be obtained. Despite a higher sensitivity for the identification of perivalvular abscesses with TEE, these can be missed in individuals with poor image quality, such as those with mitral annular calcifications or mechanical valves. In addition, abscesses are more difficult to identify prior to their cavitation, in which case repeat imaging may be warranted [16].

A recent systematic review and meta-analysis concluded that multiphase cardiac CT had a higher sensitivity (87%) than TEE (69%) for detecting abscesses and pseudoaneurysms. Since they are localized to the mitral-aortic intervalvular region, they are anatomically better visualized using CT. TEE has a higher sensitivity in detecting vegetations and all other complications of infective endocarditis when compared to CT [17].

Treatment includes targeted antimicrobial therapy for six weeks, followed by surgical repair/replacement of the valve. There have been reports of management with medical therapy alone when the abscess is less than 1 cm in patients without conduction complications or valvular insufficiencies. Very close serial TEE monitoring is required in these patients [18,19]. The aim of surgery is to both eradicate the infection and correct hemodynamic abnormalities. Valve replacement is usually essential but is heavily dependent on the extent of destruction of the surrounding supporting structures. Aortic homografts are now available to reconstruct the aorta [20].

## Conclusions

The case we report is unique, as SAFJ was the presenting feature in a patient with infective endocarditis in an immunocompetent healthy adult with no known prior predisposing conditions. The causative organism was also identified as *Streptococcus sanguinis*, which is an unusual pathogen to cause SAFJ. Initial workup (including TTE and TEE) could not identify the source of infection as aortic valve infective endocarditis in this relatively young individual. The findings, however, did increase the suspicion of infective endocarditis given severe eccentric aortic regurgitation in the setting of a bicuspid aortic valve. His progressive conduction abnormalities lowered the threshold for further imaging, eventually aiding in the identification of perivalvular abscess on cardiac CT. We would like to stress the need for re-imaging in such patients due to delay in cavitation and pseudoaneurysm formation, which can result in early false negatives. It is important to remember that using TEE and cardiac CT together can increase the sensitivity of diagnosing abscesses or pseudoaneurysms. Most patients with perivalvular abscesses require early surgical intervention and, therefore, further underscores the importance of retaining a high index of suspicion despite previous negative results.
